# Venous Diversion Surgery Revisited: A Baffling Situation

**DOI:** 10.7759/cureus.1320

**Published:** 2017-06-06

**Authors:** Jessica R Klassen, Davinder S Jassal, Brett Memauri, Malek Kass, James W Tam, Jonathan Windram, David Ross, Nasir Shaikh

**Affiliations:** 1 Department of Internal Medicine, University of Manitoba; 2 Department of Radiology, University of Manitoba; 3 Section of Cardiology, St. Boniface Hospital, University of Manitoba; 4 Department of Internal Medicine, University of Alberta; 5 Department of Surgery, University of Alberta

**Keywords:** echocardiography, computed tomography, cardiac mri, cardiac catheterization, congenital heart disease

## Abstract

With the increasing number of survivors with congenital heart disease (CHD) reaching adulthood, it is important for the clinician to be familiar with the various surgical options performed in this growing patient population. We describe the case of a 65-year-old female who presented with hypoxia and right-to-left shunting following a surgical repair of an atrial septal defect (ASD) secundum and anomalous pulmonary veins with a partial atrial diversion procedure in childhood. The use of multimodality cardiovascular imaging using echocardiography, computed tomography, magnetic resonance imaging, and invasive cardiac catheterization was complementary in the preoperative diagnosis and management of this unique baffling situation.

## Introduction

With the increasing number of survivors with congenital heart disease (CHD) reaching adulthood, it is important for the clinician to be familiar with the various surgical options performed in this growing patient population. Taking into account the complexity and limitations of clinical assessment in adult CHD, multimodality cardiac imaging techniques play an essential role in the complete evaluation of patients with this disease. 

## Case presentation

**Figure 1:**
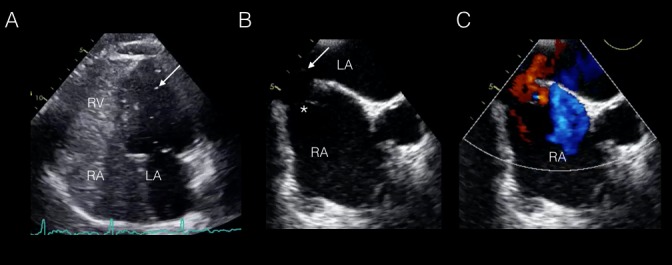
Multimodality Cardiac Imaging with Transthoracic and Transesophageal Echocardiography (A) An apical four chamber view on transthoracic echocardiography demonstrating a grade 1 intracardiac shunt following the administration of agitated saline contrast (arrow); (B-C) A bicaval view on transesophageal echocardiography without and with color Doppler demonstrating bidirectional flow across the inferior sinus venosus defect (arrow) with a prominent Eustachian valve (*). LA:, left atrium; RA: right atrium; RV: right ventricle

A 65-year-old female presented with transient left upper extremity weakness, slurred speech, and a two-year history of progressive dyspnea on minimal exertion. The past medical history was notable for a previous repair of a “hole in the heart” at the age of 11 and a non-debilitating stroke in the third decade of life. Cardiothoracic examination demonstrated mild clubbing of the distal extremities. Arterial blood gas analysis revealed a PaO_2_ of 50 mm Hg on room air. Transthoracic echocardiography (TTE) confirmed right ventricular dilatation with evidence of a grade 1 intra-cardiac shunt following the administration of agitated saline contrast through the right brachiocephalic vein (Figure 1A). Transesophageal echocardiography (TEE) demonstrated a defect in the inferior portion of the interatrial septum (IAS) with bi-directional flow on color Doppler from the inferior vena cava (IVC) to the left atrium (LA). Additionally, TEE confirmed adherence of the Eustachian valve to the IAS (Figure 1B-C). 

**Figure 2:**
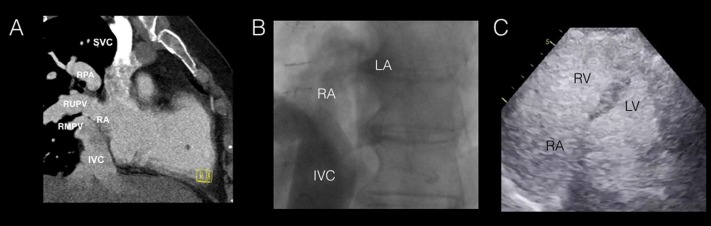
Multimodality Cardiac Imaging with Computed Tomography, Catheterization, and Echocardiography (A) Contrast-enhanced paracoronal computed tomographic image confirming the anomalous drainage of the right superior and right middle pulmonary veins into the right atrium; (B) IVC imaging during cardiac catheterization demonstrated filling of the right and left atria with the suggestion of an inferior defect in the interatrial septum; (C) An apical four chamber view on transthoracic echocardiography demonstrating significant opacification of both right and left atria following the administration of agitated saline contrast in the pigtail catheter within the IVC. IVC: inferior vena cava; LA: left atrium; LV: left ventricle; RA: right atrium; RMPV: right middle pulmonary vein; RPA: right pulmonary artery; RUPV: right upper pulmonary vein; RV: right ventricle; SVC: superior vena cava

Computed tomography (CT) and cardiac magnetic resonance imaging (CMR) confirmed evidence of anomalous right superior and middle pulmonary veins emptying into the right atrium with a Qp/Qs of > 2.0 (Figure 2A). A preoperative cardiac catheterization demonstrated no evidence of obstructive coronary artery disease. Following the injection of Omnipaque and agitated saline contrast using a pigtail catheter within the IVC, there was evidence of significant opacification of both the right and left atria (Figure 2B-C). 

## Discussion

We were unable to obtain the cardiovascular surgeon's original operative report on this patient from 1961 as the medical records had been destroyed. At the time of repeat open heart surgery, we surmised that the original pathology was a secundum ASD with separate unrelated anomalous right upper and middle pulmonary veins. The Eustachian valve had been sutured to the atrial tissue, into the inferior rim of the fossa ovalis, such that the IVC entered to the left of the atrial septum and was completely committed to the LA. Several small to medium-sized fenestrations were seen at the site of prior ASD repair, likely as a result of the degeneration at the prior repair site, leading to interatrial mixing. Remote from the ASDs, there were anomalous right upper and middle pulmonary veins entering the right atrium (RA) just below the entrance of the right superior vena cava. At surgery, using cardiopulmonary bypass (CPB) and cardioplegic arrest, the interatrial septum was completely excised. A very large patch of bovine pericardium was sutured around the orifice of the IVC and brought up along the right lateral wall of the RA, redirecting IVC flow into the right atrium and directing all pulmonary venous return to the LA. The suture line was continued superiorly and the patch was plicated in two separate spots to reduce redundancy and bulkiness, leading to unobstructed flow from the IVC to the RA and unobstructed pulmonary venous return to the LA through the newly created interatrial baffle.

Although repair of an ASD is a safe and routine procedure, the inadvertent diversion of IVC flow into the LA may lead to increased patient morbidity and mortality. Risk factors include the presence of a large ASD secundum, low-lying ASD, or inferior sinus venosum defect [1-3]. This case illustrates an uncommon complication that can occur during the surgical repair of a large secundum ASD with no inferior rim, with a clinical presentation of cyanosis, hypoxia, and chest discomfort [1-3]. In this case, the atrial tissue had been sutured to the Eustachian valve which had been mistaken by the surgeon as an inferior rim. In the era of the original repair, intraoperative cardiac imaging was not available to guide the surgeon to avoid this error, but with modern day imaging, we were able to clearly review the anatomy post-repair and to alter the management of the patient. 

## Conclusions

This unique case illustrates the importance of a multidisciplinary approach using complementary cardiac imaging in the management of complex congenital heart disease. As with all individuals who transition into the adult CHD setting, the clinician needs to be familiar with the various surgical procedures performed in this unique patient population.
